# Is It Safe to Restart Antivascular Endothelial Growth Factor Therapy in Patients with Renal Cell Carcinoma after Cardiac Ischemia?

**DOI:** 10.1155/2015/817578

**Published:** 2015-10-13

**Authors:** Bo Zhao, Laura S. Wood, Karen James, Brian I. Rini

**Affiliations:** ^1^Department of Hematology and Oncology, Cleveland Clinic, 9500 Euclid Avenue R35, Cleveland, OH 44195, USA; ^2^Department of Cardiovascular Medicine, Cleveland Clinic, 9500 Euclid Avenue J3-4, Cleveland, OH 44195, USA

## Abstract

Agents targeting vascular endothelial growth factor (VEGF) represent active drugs in treating patients with advanced renal cell carcinoma (RCC). Studies have shown that sunitinib and axitinib can be associated with cardiac toxicity. Whether these agents should be restarted in patients who experience cardiac ischemia remains uncertain. Here, we present three patients with metastatic RCC who restarted sunitinib or axitinib after intervention of active ischemic cardiac disease without causing subsequent relevant cardiac events. This experience suggests that these agents can be continued after management of cardiac ischemia.

## 1. Introduction

The development of therapeutic agents targeting vascular endothelial growth factor (VEGF) represents a major advancement in treating patients with advanced renal cell carcinoma (RCC). The beneficial effects of these agents are balanced against acute and chronic toxicities, including cardiac toxicity. Bevacizumab, a recombinant humanized monoclonal antibody that targets VEGF, has been reported to be associated with an increased risk of cardiac events [1, 2]. The rate of cardiac ischemia/infarction was 1% (5/366) in a phase III study in patients receiving bevacizumab plus interferon alfa versus 0% in patients receiving interferon alfa alone, and the rate of left ventricular dysfunction was <1% (2/366) in patient receiving bevacizumab plus interferon alfa versus 0% in patients receiving interferon alone [1]. In a meta-analysis conducted in 4,617 patients with colorectal cancer, liver cancer, and RCC treated with bevacizumab-based therapy from 7 randomized controlled trials, ischemic heart disease was 1.7% (41/2417) in patients receiving bevacizumab versus 0.6% (13/2200) in patients receiving control therapies, with a calculated relative risk of 2.49 (95% CI 1.37–4.52) [2]. Tyrosine kinase inhibitors (TKIs) such as sunitinib, axitinib, and sorafenib target the VEGF receptor. Cardiac ischemia in patients with RCC was not specifically reported from phase III studies for sunitinib or sorafenib [3–5]. In an observational study, 33.8% (25/74) patients receiving sunitinib or sorafenib experienced a cardiovascular event, including 7 patients who required coronary angiography [6]. In a recent analysis of pooled data from clinical trials in 672 patients with metastatic RCC and 1,304 patients with advanced solid tumors, 33 patients (1.7%) developed myocardial infarction (MI) on axitinib [7]. However, whether TKIs should be restarted in patients who receive successful intervention for cardiac ischemia remains uncertain, as such patients on clinical trials would have discontinued therapy. Here, we present three patients with metastatic RCC who safely restarted anti-VEGF TKIs after intervention of active ischemic cardiac disease.

## 2. Case Presentation

### 2.1. Case 1

A 55-year-old man was diagnosed with recurrent RCC in mediastinal lymph nodes one year after right partial nephrectomy for a Fuhrman grade 3, clear cell tumor. He was started on sunitinib 50 mg once daily, in cycles of 4 weeks on and two weeks off schedule on the phase III clinical trial of sunitinib versus interferon-alfa as a first-line systemic therapy for patients with metastatic RCC. The dose was reduced to 37.5 mg after 11 cycles due to multiple recurrent grade 2 toxicities including fatigue, hand-foot syndrome, arthralgias, and myalgias. He had no known underlying coronary artery disease (CAD) at enrollment, and risk factors for CAD included a 25 pack-year smoking history, family history of premature CAD (brother < 55 years), and baseline hypertension of 140/85 mm Hg on moexipril 15 mg once daily. He developed grade 2 hypertension on sunitinib, which improved to 130/70 mm Hg after adding 25 mg of hydrochlorothiazide once daily. His creatinine was 1.3 mg/dL upon initiation of sunitinib with a calculated GFR of 61 mL/min/1.73 m^2^, which remained relatively stable throughout his disease course. Five years later, he was noted to have developed coronary artery atherosclerotic calcifications on computed tomography scans. There were no symptoms to suggest angina at that time, and a stress test showed no evidence of ischemia. However, 14 months later, he developed acute onset chest pain and was found to have ST elevation indicative of myocardial infarction (MI) in the inferior wall. An emergent cardiac catheterization revealed 100% stenosis in right coronary artery (RCA) and 80% stenosis in left anterior descending artery (LAD). He received staged percutaneous coronary intervention (PCI) with two bare metal stents placement in the RCA and an everolimus-eluting stent in the LAD 10 days later. Sunitinib was restarted at the same dose after a holiday of ninety days when scans showed disease progression. An echocardiogram one day after the MI showed left ventricular ejection fraction (LVEF) of 50–55%, with hypokinesis of the posteriolateral and septal myocardium; a repeat echocardiogram 2 weeks before restarting sunitinib showed stable findings. He remained on sunitinib for another two years until disease progression, when he was switched to axitinib. The patient has been on axitinib for 2 years, currently on 8 mg twice daily. No further cardiac ischemia or other cardiac events have occurred.

### 2.2. Case 2

A 52-year-old man was diagnosed with biopsy-proven, recurrent RCC in left adrenal gland one year after radical left nephrectomy. The primary tumor was Fuhrman grade 4, clear cell histology. He was started on axitinib through a randomized, double-blind, phase II study of axitinib with or without dose titration in patients with metastatic renal cell carcinoma. He had no known CAD at baseline, and risk factors for CAD included mixed type hyperlipidemia (normal cholesterol level on diet control) and a long-standing hypertension diagnosed at the age of 25. His blood pressure readings at baseline were 130/70 mm Hg on candesartan-hydrochlorothiazide 32 mg/25 mg. His creatinine was 1.2 mg/dL upon initiation of axitinib, and calculated GFR was 61 mL/min/1.73 m^2^, which remained relatively stable throughout his disease course. He then developed grade 3 hypertension on axitinib, which improved to 120/70 mm Hg after adding once daily 5 mg amlodipine. He achieved an objective complete response per RECIST criteria v1.0 six months after the initiation of axitinib. Three years and 9 months later, he developed exertional chest pain. Axitinib was held, and two days later he underwent a cardiac catheterization evaluation, which revealed 99% stenosis of ostial/proximal LAD, 70% stenosis of proximal left circumflex artery (LCx), and 90% stenosis in mid and distal RCA. EKG was sinus rhythm without evidence of ischemia, and LVEF was 60% on echocardiogram. He received successful staged PCI with placement of drug eluting stents (DES) in LAD and left circumflex artery (LCx), followed by mid and distal RCA with two more DES 4 days later. Axitinib was restarted 3 days after the second PCI at the same dose and schedule. Repeat lipid panel showed elevated cholesterol level of 345 mg/dL and triglyceride level of 362 mg/dL, ezetimibe-simvastatin 10–20 mg was added, and cholesterol and triglyceride levels were then normalized. The patient has remained on axitinib for one and half years since his cardiac ischemia with continued complete response. No subsequent cardiac ischemia or other cardiac events have been reported. A repeat echocardiogram one year later showed LVEF of 56 ± 5% with normal systolic function.

### 2.3. Case 3

A 63-year-old man presented with low back pain and was found to have a vertebral soft tissue mass in the second lumbar vertebra (L2), a right kidney 7 cm contrast-enhancing tumor, multiple subcentimeter lung lesions, and bilateral adrenal nodules. Needle biopsy of the kidney tumor was consistent with RCC. He underwent palliative radiotherapy to the L2 vertebral lesion and debulking right nephrectomy, with pathology showing Fuhrman grade 3, clear cell RCC. He was started on front line axitinib through a randomized, double-blind phase II study of axitinib with or without dose titration in patients with metastatic renal cell carcinoma. At that time, he did not have symptoms to suggest active CAD, and risk factors for CAD included mixed type hyperlipidemia (normal cholesterol level on simvastatin 20 mg), a former 15 pack-year smoking history (quitted 30 years earlier), and hypertension. His blood pressure readings at baseline were 119/71 mm Hg on metoprolol tartrate 50 mg and hydrochlorothiazide 25 mg. His creatinine level was 1.68 mg/dL with a calculated GFR of 41 mL/min/1.73 m^2^, which remained relatively stable throughout his disease course. Amlodipine 10 mg was added for grade 3 hypertension that developed on axitinib, and his blood pressure improved to 120/70 mm Hg. Two years and 5 months later, he experienced worsening recurrent chest pain 3 days after holding axitinib for grade 2 anorexia. EKG showed borderline T wave inversion in the anterior leads, and cardiac enzymes were within normal limits. A thallium stress test showed mild reversible changes involving the inferior wall. Further cardiac catheterization showed stenosis involving multiple vessels, including 95% of ostial RCA, 90% of mid and inferior segment of the posterolateral branch, 50% of distal left main, 50–60% of proximal and mid LAD, 90% of distal LAD, and 80% mid circumflex, and diffuse stenosis in distal LAD and mid circumflex. His calculated LVEF was 80% on stress test, and left ventricular systolic function was normally evaluated by ventriculogram. He was evaluated by cardiovascular surgery and deemed not a candidate for surgical intervention. Subsequently, he underwent staged PCI: stage I intervention with 4 DES was placed in ostial RCA, superior branch posterior left ventricular branch, inferior portion posterior left ventricular branch, and posterior descending artery. Stage II intervention with 2 DES was placed in mid-LAD and the circumflex 10 days later. Axitinib was held until 12 days after the stage II PCI. Metoprolol tartrate 50 mg and fenofibrate micronized 48 mg were added; his hypertriglyceridemia was improved from 408 mg/dL to 241 mg/dL. However, he subsequently experienced two more episodes of angina 6 months and 1 year and 10 months after the first angina and received 1 DES for a proximal 95%–99% LAD stenosis and 1 DES for a mid-LAD 95% stenosis, respectively. Axitinib was interrupted for 12 days and 24 days for these events, respectively. Both repeated echocardiograms after these two PCI interventions showed normal sized right and left ventricles with normal systolic function. He had disease progression after the third episode of angina, and the dose of axitinib was increased to 5 mg twice daily from 3 mg twice daily, which resulted in regression of his RCC. He is currently on axitinib 5 mg for an additional year without recurrent cardiac events.  Table 1 summarizes the major clinical features of the three cases.

## 3. Discussion

The role of angiogenesis in ischemic heart disease has been controversial. While therapeutic angiogenesis has been thought to promote revascularization in ischemic cardiovascular disease, data from clinical trials have been inconclusive so far [8]. In the NORTHERN Trial, 93 patients with refractory Canadian Cardiovascular Society class 3 or 4 anginal symptoms were randomized to receive VEGF plasmid DNA or placebo (buffered saline) delivered via the endocardial route using an electroanatomical guidance catheter. There was no difference between the VEGF-treated and the placebo groups with respect to the change in myocardial perfusion, or improvements in exercise treadmill time and anginal symptoms [8]. On the other hand, studies suggest that neovascularization contributes to the growth of atherosclerotic lesions and is a key factor in plaque destabilization leading to rupture, which may cause acute coronary syndromes [9]. Although ischemic cardiac disease appears to be increased in patient receiving anti-VEGF TKIs [1–3], our patients did not experience ischemic cardiac events for at least two years after starting sunitinib or axitinib. Moreover, no patients experienced recurrent ischemia immediately after restarting these agents. This is consistent with the observation from another study, in which all 7 patients who experienced cardiac ischemia on sunitinib or sorafenib resumed the same TKI after medical intervention without causing subsequent clinically relevant cardiac events [6]. Another concern is that anti-VEGF TKIs may affect ventricular systolic function, as a normal angiogenic response has been proposed to be necessary to maintain an adequate response of cardiomyocytes to pressure load [10]. In fact, a decline in ejection fraction was reported in 10% patients receiving sunitinib in a randomized phase III study, as opposed to 3% in patients receiving interferon alfa [3]. No decreased ejection fraction was reported in the pooled analysis of axitinib [7]. For our patients, none of them had clinical evidence of congestive heart failure, with documented preserved and stable LVEF after resuming the same TKIs. Taken together, our experience suggests that after effective intervention of coronary artery stenosis, sunitinib or axitinib can be safely restarted without causing decline of ventricular systolic function or subsequent increased risk of recurrent cardiac ischemia. The suitability and timing of the resumption of treatment should be evaluated case by case, based on the clinical conditions of the individual patient.

## Figures and Tables

**Table 1:** Summary of ischemic events and interventions on anti-VEGF TKIs.

Case	1	2	3
Age at diagnosis (years)	54	51	63
TKI during cardiac event	Sunitinib	Axitinib	Axitinib
Ischemic event	MI	Angina	Angina
Intervention	Stent ×3	Stent ×4	Stent ×6
Known underlying CAD	No	No	No
Days on TKI before the event	2256	1384	885
Duration of break during cardiac event (days)	77	9	28
Days on the same TKI after the event	721	450	1080
LVEF after the event (%)	55	60	55
CHF at any time	No	No	No
Subsequent ischemia	No	No	Yes

VEGF: vascular endothelial growth factor; TKI: tyrosine kinase inhibitor; MI: myocardial infarction; CAD: coronary cardiac disease; CABG: coronary artery bypass graft; LVEF: left ventricular ejection fraction; CHF: congestive heart failure.

## References

[1] Rini B. I., Halabi S., Rosenberg J. E. (2010). Phase III trial of bevacizumab plus interferon alfa versus interferon alfa monotherapy in patients with metastatic renal cell carcinoma: final results of CALGB 90206. *Journal of Clinical Oncology*.

[2] Chen X.-L., Lei Y.-H., Liu C.-F. (2013). Angiogenesis inhibitor bevacizumab increases the risk of ischemic heart disease associated with chemotherapy: a meta-analysis. *PLoS ONE*.

[3] Motzer R. J., Hutson T. E., Tomczak P. (2007). Sunitinib versus interferon alfa in metastatic renal-cell carcinoma. *The New England Journal of Medicine*.

[4] Motzer R. J., Hutson T. E., Cella D. (2013). Pazopanib versus sunitinib in metastatic renal-cell carcinoma. *The New England Journal of Medicine*.

[5] Escudier B., Eisen T., Stadler W. M. (2007). Sorafenib in advanced clear-cell renal-cell carcinoma. *The New England Journal of Medicine*.

[6] Schmidinger M., Zielinski C. C., Vogl U. M. (2008). Cardiac toxicity of sunitinib and sorafenib in patients with metastatic renal cell carcinoma. *Journal of Clinical Oncology*.

[7] Rini B. I., Escudier B., Hariharan S. (2015). Long-term safety with axitinib in previously treated patients with metastatic renal cell carcinoma. *Clinical Genitourinary Cancer*.

[8] Stewart D. J., Kutryk M. J. B., Fitchett D. (2009). VEGF gene therapy fails to improve perfusion of ischemic myocardium in patients with advanced coronary disease: results of the NORTHERN trial. *Molecular Therapy*.

[9] Khurana R., Simons M., Martin J. F., Zachary I. C. (2005). Role of angiogenesis in cardiovascular disease: a critical appraisal. *Circulation*.

[10] Shiojima I., Sato K., Izumiya Y. (2005). Disruption of coordinated cardiac hypertrophy and angiogenesis contributes to the transition to heart failure. *Journal of Clinical Investigation*.

